# Factors Associated with Comfort Discussing PrEP with Healthcare Providers among Black Cisgender Women

**DOI:** 10.3390/tropicalmed8090436

**Published:** 2023-09-07

**Authors:** Whitney C. Irie, Anais Mahone, Raja Nakka, Musie Ghebremichael

**Affiliations:** 1 Boston College School of Social Work, Chestnut Hill, MA 02467, USA; 2 The Fenway Institute, Fenway Health, Boston, MA 02215, USA; 3 School of Social Work, Rutgers University, New Brunswick, NJ 08901, USA; 4 The Ragon Institute of MGH, MIT, and Harvard, Cambridge, MA 02139, USAmusie_ghebremichael@dfci.harvard.edu (M.G.); 5 Department of Medicine, Harvard Medical School, Cambridge, MA 02215, USA

**Keywords:** preexposure prophylaxis, human immunodeficiency virus (HIV), stigma, Black women

## Abstract

Preexposure prophylaxis (PrEP) for HIV prevention uptake remains low among Black cisgender women in the United States, despite their disproportionate HIV burden. This study aimed to examine factors associated with Black women’s comfort discussing PrEP with healthcare providers and its link to their interest in PrEP use. A cross-sectional survey was conducted with a national sample of 315 Black cisgender women. Descriptive statistics and logistic regression models were utilized for data analysis. The results showed that 79% of Black women felt comfortable discussing PrEP with their healthcare provider. Age, recent healthcare provider visit, interest in PrEP, and positive social norms were associated with increased odds of comfort in discussing PrEP, while anticipated PrEP disapproval and stigma were associated with decreased odds. Older age was related to greater comfort, potentially due to increased familiarity and self-efficacy in discussing sexual health. Recent healthcare utilization indicated positive provider relationships, facilitating discussions about sexual health. Anticipating support from social networks positively influenced comfort levels. Conversely, PrEP-related stigma and anticipated disapproval were barriers to comfort. These findings highlight the importance of provider–patient communication and social support in facilitating PrEP engagement among Black cisgender women. Interventions should consider age-appropriate strategies and address structural and provider biases to improve PrEP discussions and promote HIV prevention.

## 1. Introduction

Despite its efficacy, preexposure prophylaxis (PrEP) for HIV prevention uptake remains low among women in the United States (US) [1]. Women comprise 20% of new HIV infections in the U.S., but only 4.7% of those are prescribed PrEP [2]. Black cisgender women continue to be disproportionately impacted by HIV in comparison to women of other racial and ethnic groups [1]. Yet, black women remain largely unaware of PrEP [3,4,5]; those with PrEP awareness prefer to receive PrEP from their primary care provider [6]. However, rates of PrEP utilization among Black women remain low. There is a need to examine the patient–provider relationship as critical for PrEP engagement for Black women.

As indicated in previous studies, although most women had not heard about PrEP from their healthcare provider, most viewed their provider as a preferred source of PrEP information and considered their provider as the ideal source of PrEP care [7]. Also consistent with the literature, women viewed PrEP as an important component of HIV prevention care and would be more likely to consider PrEP uptake if PrEP was discussed and prescribed by their healthcare providers within trusted venues for routine health services, such as sexual health clinics and primary care facilities [7,8]. Further, most women preferred PrEP to be offered in conjunction with other general health services or integrated into drug treatment programs rather than seeking a specialist to prescribe the medication [9].

Communication about PrEP in the primary care settings is critical toward reducing racial disparities in outcomes among women; however, US women indicated in a qualitative study that providers rarely asked about behaviors such as sexual practices and drug use related to HIV acquisition [10,11]. Additionally, women felt that if they disclosed sexual behaviors to their providers they might experience judgment and disparaging treatment [11]. Racial disparities in healthcare may further complicate communication. One study found that Black women had significantly higher levels of mistrust towards healthcare personnel than White women, which decreased their comfort in discussing PrEP with a healthcare provider despite a greater interest in initiating PrEP [12]. There is a growing yet incomplete body of literature using survey data to identify factors associated with Black women’s comfort discussing PrEP with their healthcare providers. The current study contributes to this body of literature with added examination of sociocultural factors including social norms and stigma.

It is within this context of social barriers (i.e., anticipated stigma/discomfort) to discussing PrEP that this study seeks to use a national sample of survey data to examine Black women’s comfort discussing PrEP with their healthcare providers and its association with interest in using PrEP for HIV prevention.

## 2. Materials and Methods

### 2.1. Methods

Please note that the methods described below have also been reported elsewhere [13]. We conducted a cross-sectional survey using the Qualtrics panel research service to obtain a national sample of adult Black cisgender women. This sampling approach is an established method in HIV research [14] including studies focused on HIV and PrEP among Black Americans [15,16,17,18,19]. Sampling and survey design methods have been previously described and referenced in a previous publication [13].

We collected self-reported sociodemographic details encompassing age, relationship status, level of education, household income, status of health insurance, and employment situation. To gauge involvement with healthcare, participants were asked whether they had visited a medical professional within the preceding 12 months and if they had refrained from seeking healthcare during the same period due to financial reasons. Aspects of sexual history were examined by inquiring about the number of sexual partners and the frequency of condom usage in the last 6 months. Respondents were also asked if they had engaged in transactions involving sex for money, drugs, housing, or other items, if they had an HIV test in the past year, and if they had ever received treatment for a sexually transmitted infection (STI) in their lifetime. Participants’ concerns regarding the potential acquisition of HIV were assessed using a single question: “Do you experience any concerns about the possibility of contracting HIV?” answered with “yes” or “no”.

Respondents received PrEP education via a CDC PrEP infographic and were asked: “Before today have you heard of PrEP?” Respondents were then asked: “Are you interested in using PrEP to prevent HIV infection?” [yes/no].

We used the validated PrEP anticipated stigma subscales to assess PrEP stigma [20]. Of the two subscales, the PrEP-user stereotypes subscale measures perceived cultural associations with PrEP (Cronbach’s alpha = 0.85), and the PrEP disapproval by others subscale (Cronbach’s alpha = 0.81) measures expected judgments from others for using PrEP.

A single item assessed comfort discussing PrEP by asking, “Would you feel comfortable discussing PrEP with your healthcare provider/doctor?” [yes/no].

### 2.2. Statistical Analysis

Descriptive measures (i.e., mean, standard deviation, frequencies, and percentages) were used to summarize the data. Fisher exact and Wilcoxon rank sum tests were used to compare categorical and continuous variables between women who did and did not feel comfortable discussing PrEP with their healthcare providers. Univariate and multivariate logistic regression models were utilized to assess the predictors of comfort in discussing PrEP with providers. The maximum likelihood method was used to estimate model parameters, and significance was tested using the Wald test statistic. Statistical analyses were performed using the R package version 4.0.3 and SAS software version 9.4 (SAS Institute, Cary, NC, USA). All *p*-values were 2-sided and considered statistically significant if <0.05.

## 3. Results

### 3.1. Sample Characteristics

We analyzed a nationwide sample comprising 315 Black cisgender women. The details describing this group are outlined in Table 1. The median age stood at 29 years, with an interquartile range of 22 to 35 years. Geographically, the distribution of participants mirrored that of Black Americans throughout the United States. A significant portion (55.2%) of the respondents lived in the Southern region during the survey period. A substantial majority of participants (50.5%) possessed some level of college education, while a notable proportion were employed (78.1%) and held health insurance coverage (88.6%). Moreover, 34.6% of individuals reported an annual income of $40,000 or higher. Over the course of the preceding year, 88.3% of participants had sought medical attention from a healthcare provider, but 26.3% refrained from doing so due to financial concerns. In terms of relationship status, slightly more than half (52.7%) were in a partnership, and 12.4% acknowledged having either inconsistent or no condom use along with involvement in two or more sexual partnerships. In a broader context, more than half (57.8%) expressed apprehension regarding the possibility of contracting HIV infection.

### 3.2. Comfort Discussing PrEP with Providers

Our outcome of interest, comfort discussing PrEP with providers, was dichotomized. In the sample, 79% of Black women felt comfortable talking to their healthcare provider about PrEP.

When comparing the median values for age and the PrEP scales between comfortable and not comfortable discussing PrEP with providers about PrEP, there were significant differences in median values among all the PrEP scales between the two groups (Table 2).

Black women who were not comfortable discussing PrEP with their provider had higher median PrEP disapproval (Median = 7, IQR = 6–9, *p* < 0.0001) and PrEP-user stereotypes (Median = 12, IQR = 9–15, *p* < 0.001) scores compared to Black women who were comfortable talking to their provider about PrEP. Black women who were comfortable discussing PrEP with their providers had higher overall positive subjective norms (Median = 12, IQR = 10–14, *p* < 0.0001), injunctive (Median = 6, IQR = 6–8, *p* < 0.0001), and descriptive norms (Median = 6, IQR = 5–7, *p* < 0.0001) scores than women who were not comfortable discussing PrEP with their providers. A significantly larger proportion of Black women who expressed interest in PrEP were comfortable discussing PrEP with their providers (Table 3., *p* < 0.0001). There were no significant differences found among other factors in the study.

**Figure 1:**
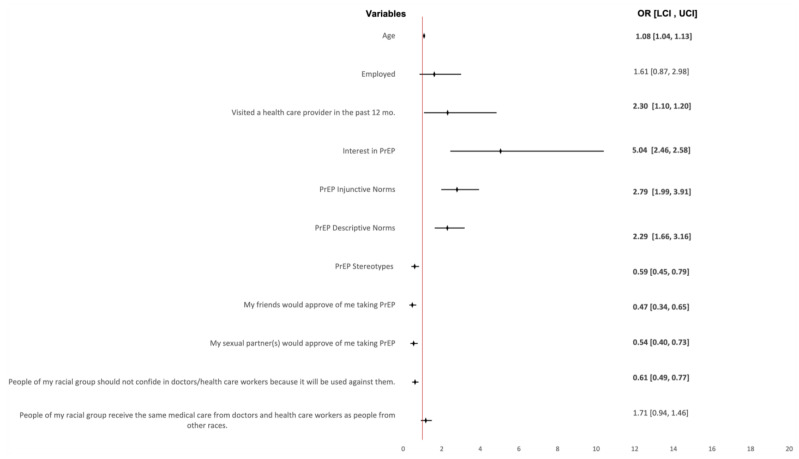
Forest plot representing odds ratios of comfort discussing PrEP with provider with corresponding 95% confidence intervals (CI).

Figure 1 displays the odds of having comfort discussing PrEP with a healthcare provider together with their corresponding 95% confidence intervals. Anticipated PrEP disapproval from friends/peers (OR = 0.47, 95% CI: 0.47–0.34; *p*-value < 0.0001), sexual partners (OR = 0.54, 95% CI: 0.40–0.73; *p*-value < 0.0001), and anticipated PrEP stereotypes (OR = 0.59, 95% CI: 0.44–0.79; *p*-value ≤ 0.0001) were associated with decreased odds of having comfort discussing PrEP with a healthcare provider. However, age (OR = 1.08, 95% CI: 1.03–1.13; *p*-value ≤ 0.0001), having seen a provider in the past 12 months (OR = 2.30, 95% CI: 1.10–4.81; *p*-value = 0.027), interest in taking oral PrEP (OR = 5.04, 95% CI: 2.46–10.33; *p*-value < 0.0001), injunctive norms (OR = 2.79, 95% CI: 1.99–3.90; *p*-value < 0.0001) and descriptive norms (OR = 2.29, 95% CI: 1.66–3.16; *p*-value < 0.0001) were associated with increased odds of having comfort discussing PrEP with a healthcare provider.

## 4. Discussion

Patient–provider communication during the clinical encounter is key at every step along the PrEP care continuum; of most importance is communication during the PrEP initiation stage. Using a national sample of Black cisgender women, this study contributes one of the first quantitative examinations of the demographic and social factors associated with Black women’s comfort with discussing PrEP with a healthcare provider. Most Black women in our sample were comfortable discussing PrEP with their provider, although nearly 20% of our sample were not comfortable having this type of discussion with their provider. We found that comfort with discussing PrEP with a healthcare provider is influenced by several factors: older age, individuals who had recently seen a healthcare provider, and anticipating positive social norms and support around PrEP use. Conversely, we found that anticipated disapproval and PrEP-related stigma were significant factors associated with lower odds of comfort in discussing PrEP with healthcare providers.

In our sample, older age was associated with comfort in discussing PrEP with their healthcare provider. Age can influence the level of comfort and satisfaction in healthcare interactions; in a sample of Black and Hispanic women, results show that younger age was associated with discomfort with discussing sexual health topics with healthcare providers [21]. Further, other studies have found that age is negatively associated with receiving HIV prevention services from providers such as HIV testing due to lower HIV risk perception [22,23]. In our sample of Black women, age may be associated with greater familiarity with healthcare providers and systems, and for some Black women, as they age, they may feel greater autonomy and self-efficacy discussing sexual health and HIV prevention with healthcare providers. Young Black women face unique challenges in healthcare due to sociocultural barriers, including limited communication about sexual topics [24,25,26]. Recent studies highlight the lack of sexual and reproductive health education and discussions Black women receive from their parents [24]. While benefiting from obtaining sexual health information from providers, qualitative studies have shown that Black women often feel humiliated and invalidated when providers doubt their experiences [26]. Insufficient support and information due to provider disengagement and provider bias leaves young Black women ill-equipped to engage effectively in their sexual and reproductive healthcare [25]. Our finding points to the necessity of PrEP interventions that utilize a life course approach [27,28], recognize the intergenerational impact of structural trauma, and employ strategies that are age-appropriate to improve communication and rapport-building between younger Black women and their healthcare providers.

In our sample, we found that individuals who had recently seen a healthcare provider were more likely to feel comfortable discussing PrEP. This could indicate what similar studies have shown: continued or recent engagement in preventative healthcare is associated with high satisfaction with healthcare providers and health systems [29]. This positive and established relationship with a healthcare provider has the potential to facilitate easier conversations about sexual health [30,31]. Other studies have shown that recent healthcare utilization is influenced by provider trust among populations impacted by HIV, particularly Black Americans [32]. In addition, results from another study have shown that Black women who experience positive encounters with family planning providers report trusting their providers, having greater comfort asking questions and increased confidence in navigating the healthcare system [25]. And Black women who reported greater mistrust had lower comfort discussing PrEP with their providers [12]. While we did not collect data regarding whether the respondents have a primary care provider, recently visiting a healthcare provider could indicate healthcare engagement and utilization further highlighting the importance of positive provider interactions in facilitating informative discussions about PrEP for Black women.

Anticipating positive social norms and support around PrEP was also associated with comfort in discussing PrEP with a healthcare provider. This suggests that individuals who perceive a supportive social environment regarding PrEP are more likely to feel at ease discussing it with their healthcare provider. This finding highlights the social complexity of PrEP engagement for Black women. It is important that we are able to situate the decision to use PrEP among Black women within a context where social norms, support, and expectations are notable factors that influence their decisions and behaviors. This may contradict the framing of PrEP as a “woman-controlled, discrete” HIV prevention option through the recognition that although PrEP use does not require disclosure or discourse with peers, partners, family, or other social supports, anticipated or perceived support is a determining factor. This is further supported by our next findings, that anticipated disapproval and PrEP-related stigma were significant factors associated with lower odds of comfort in discussing PrEP with healthcare providers.

PrEP-related stigma including misconceptions about its effectiveness, concerns about judgment, and fears of being labeled as promiscuous or engaging in risky behavior can significantly deter Black women from seeking information and engaging in open conversations with their healthcare providers. This finding highlights the urgent need for comprehensive interventions that tackle both the individual-level and structural-level factors contributing to PrEP-related stigma. Such interventions should involve education, community engagement, and destigmatization efforts to promote accurate knowledge about PrEP and challenge harmful stereotypes. The findings of this study suggest that addressing anticipated disapproval and PrEP-related stigma should be key components of interventions aimed at improving PrEP engagement and uptake among Black women.

Findings are consistent with conclusions from other studies that social stigma surrounding HIV risk behavior, PrEP, and HIV hindered women’s openness with their providers [33,34,35]. Past experiences of judgment and the desire to avoid associated embarrassment and shame discouraged women from having candid discussions with their providers. Awareness of the stigma attached to risk behaviors, PrEP and HIV can help healthcare providers more conscientiously and skillfully introduce respectful discussions of risk and HIV prevention measures with women. Despite the stigma and associated feelings of judgment/embarrassment, women prefer that providers initiate discussions about PrEP-relevant risk behaviors rather than leaving the burden of initiating this discussion on the woman [36]. This may, in part, explain why providers who more often initiate discussions of PrEP with their patients are more likely to also prescribe PrEP to women [37].

In our study, comfort with discussing PrEP with a healthcare provider was a significant predictor of increased interest in PrEP. This association aligns with previous research emphasizing the importance of provider–patient communication in shaping sexual healthcare decisions [38,39]. In the context of HIV prevention, effective communication between healthcare providers and patients can help address misconceptions, alleviate fears and stigma, and promote the understanding of PrEP as a viable option for reducing HIV transmission risk. Providers who are well-informed about PrEP and its benefits can play a crucial role in empowering Black women to make informed decisions regarding their sexual health.

These results highlight the complex social and cultural dynamics that impact PrEP engagement and uptake among Black women, necessitating targeted interventions to address these barriers. Black women who anticipated disapproval from healthcare providers or their immediate social networks were less likely to feel comfortable discussing PrEP. This finding underscores the enduring presence of stigma surrounding HIV prevention strategies and the negative attitudes that persist within both healthcare settings and broader social circles. Addressing and challenging these negative perceptions is crucial to fostering an environment where Black women feel supported and empowered to discuss and access PrEP. Furthermore, our findings underscore the importance of provider training and education on PrEP for Black women, cultural competency, and addressing health inequities within the context of HIV prevention. Healthcare systems should prioritize efforts to improve provider–patient communication, enhance PrEP knowledge and awareness, and ensure equitable access to PrEP services for Black women who bear a disproportionate burden of HIV infections.

### Limitations

While this study provides valuable insights, it is important to acknowledge its limitations. The study relied on self-reported data, which may be subject to recall bias and social desirability bias. Additionally, the cross-sectional nature of the study limits our ability to establish causality or determine the directionality of the observed associations. Future longitudinal studies are needed to further explore the dynamics between provider–patient communication, comfort with discussing PrEP, and sustained interest and uptake of PrEP among Black women.

Despite these limitations, our study is strengthened by the partnership with a community advisory board throughout the development of the survey and incorporated their feedback on survey questions, including potential response bias, prior to survey administration. Additionally, the sample frame included a general population of Black women nationwide distinguishing it from many HIV prevention studies among high-risk women and/or women attending healthcare clinics.

## 5. Conclusions

In conclusion, our findings highlight the critical role of healthcare providers in fostering discussions about PrEP and influencing interest in PrEP among Black women. Creating a supportive and non-judgmental environment, addressing stigma and misconceptions, and providing accurate information about PrEP are essential components of HIV prevention efforts. Efforts to enhance provider training, improve communication, and reduce stigma can contribute to increased PrEP uptake and ultimately reduce the disproportionate impact of HIV on Black women in the United States.

## Figures and Tables

**Table 1:** Characteristics of respondents. (*N* = 315).

	*N* (%)
Age *Median* (*IQR*)	29 (22–35)
Age groups	
Younger than 30 years	174 (55.2)
Older than 30 years	141 (44.8)
Relationship status	
Single	149 (47.3)
In Relationship	166 (52.7)
Education	
Less than high school degree	6 (1.9)
High School/GED	150 (47.6)
Technical/Associate Degree	70 (22.2)
Bachelors or Higher	89 (28.3)
Employment	
Employed	246 (78.1)
Unemployed	69 (21.9)
Household income, USD	
Greater than equal to $40,000	109 (34.6)
Less than $40,000	206 (65.4)
Geographic region	
Midwest	72 (22.8)
Northeast	42 (13.3)
South	174 (55.2)
West	27 (8.7)
Health insurance status	
Insured	279 (88.6)
Uninsured	36 (11.4)
Visited a health care provider, past 12 months	278 (88.3)
Did not receive healthcare due to cost, past 12 months	83 (26.3)
Inconsistent or no condom use and multiple partners, past 6 months	39 (12.4)
Worry about HIV Infection	182 (57.8)
Comfort Discussing PrEP with Healthcare Provider	
Comfortable	249 (79.0)
Not Comfortable	66 (21.0)
PrEP subjective norms score *Median* (*IQR*)	12 (10–13)
PrEP injunctive norms score *Median* (*IQR*)	6 (5–7)
PrEP descriptive norms score *Median* (*IQR*)	6 (4–6)
PrEP disapproval by others subscale score *Median* (*IQR*)	6 (5–8)
PrEP-user stereotypes subscale score *Median* (*IQR*)	11 (8–14)

PrEP, preexposure prophylaxis; HIV, human immunodeficiency virus. PrEP subjective norms scale scores range from 4 to 16, with higher scores indicating greater more favorable norms. PrEP injunctive and descriptive norms subscale scores range from 2–8, with higher scores indicating more favorable norms towards PrEP. PrEP disapproval by others subscale scores range from 3 to 12, with higher scores indicating greater anticipated disapproval. PrEP-user stereotypes subscale scores range from 5 to 20, with higher scores indicating greater anticipated stereotyping. IQR, Interquartile range.

**Table 2:** Comparison of age, PrEP disapproval, PrEP user stereotypes, and PrEP related subjective norms between Black women who feel comfortable discussing PrEP with their healthcare providers about PrEP and those who did not feel comfortable discussing PrEP with their healthcare providers.

	Comfort Discussing PrEP	No Comfort Discussing PrEP	
	(*N =* 249)	(*N =* 66)	
	*Median* (*IQR*)	*Median* (*IQR*)	*p*
Age	29 (23–36)	23 (20–33)	<0.0001
PrEP disapproval by others subscale score	6 (5–7)	7 (6–9)	<0.0001
PrEP-user stereotypes subscale score	11 (8–13)	12 (9–15)	0.011
PrEP subjective norms score	12 (10–14)	9 (8–12)	<0.0001
PrEP injunctive norms score	6 (6–8)	5 (4–6)	<0.0001
PrEP descriptive norms score	6 (5–7)	5 (3–6)	<0.0001

PrEP, preexposure prophylaxis; PrEP disapproval by others subscale scores range from 3 to 12, with higher scores indicating greater anticipated disapproval. PrEP disapproval responses were anchored as follows: 1 = strongly agree, 2 = agree, 3 = disagree, 4 = strongly disagree. PrEP-user stereotypes subscale scores range from 5 to 20, with higher scores indicating greater anticipated stereotyping. PrEP subjective norms scale scores range from 4 to 16, with higher scores indicating greater and more favorable norms. PrEP injunctive and descriptive norms subscale scores range from 2–8, with higher scores indicating more favorable norms toward PrEP. *p*-values were obtained from Wilcoxon rank sum tests. IQR Interquartile range.

**Table 3:** Individual and social factors in Black women who feel comfortable discussing PrEP with their providers and those who do not feel comfortable discussing PrEP with their providers (*N* = 315).

	Comfortable	Not Comfortable	
	*N* (%)	*N* (%)	*p*
Educational level			0.166
No college degree	118 (75.6)	38 (24.4)	
Earned a college degree	131 (82.4)	28 (17.6)	
Employment			0.135
Unemployed	50 (72.5)	19 (27.5)	
Employed	199 (80.9)	47 (19.1)	
Household income, USD			0.468
Less than $40,000	160 (77.7)	46 (22.3)	
Greater than equal to $40,000	89 (81.7)	20 (18.3)	
Relationship Status			0.213
Single	113 (75.8)	36 (24.2)	
In a relationship	136 (81.9)	30 (18.1)	
Insurance Status			0.518
Uninsured	27 (75.0)	9 (25.0)	
Insured	222 (79.6)	57 (20.4)	
Visited a health care provider, past 12 months			0.031
No	24 (64.9)	13 (35.1)	
Yes	225 (80.9)	53 (19.1)	
Do you worry about HIV infection?			0.999
No	105 (78.9)	28 (21.1)	
Yes	144 (79.1)	38 (20.9)	
How interested are you in using PrEP to prevent HIV infection?			<0.0001
Very uninterested	64 (66.0)	33 (34.0)	
Somewhat uninterested	51 (77.3)	15 (22.7)	
Somewhat interested	85 (88.5)	11 (11.5)	
Very interested	49 (87.5)	7 (12.5)	
Inconsistent or no condom use and multiple sexual partners, past 6 months			0.68
No	219 (79.3)	57 (20.7)	
Yes	30 (76.9)	9 (23.1)	
Did not receive healthcare due to cost, past 12 months			0.531
No	181 (78.0)	51 (22.0)	
Yes	68 (81.9)	15 (18.1)	
Ever treated for an STI			0.169
No	84 (45.2)	102 (54.8)	
Yes	69 (53.5)	60 (46.5)	

PrEP, preexposure prophylaxis; HIV, human immunodeficiency virus; STI, sexually transmitted infection; *p*-values were obtained from Fisher exact tests.

## Data Availability

The data that support the findings of this study are available from the corresponding author, Whitney Irie, upon reasonable request.
